# Demographic Characteristics and Evolution of Catheterization Patterns Among Cancer Patients with Peripherally Inserted Central Catheters in Huangshi, China: A Single-Center Retrospective Study

**DOI:** 10.3390/healthcare14203442

**Published:** 2026-10-14

**Authors:** Jumin Xie, Xinyi Lv, Xuening Wang, Zejing Jiang, Yuxuan Xie, Changhui Ren

**Affiliations:** 1 Hubei Key Laboratory of Renal Disease Occurrence and Intervention, School of medicine, Hubei Polytechnic University, Huangshi 435003, China; lvxinyi0620@163.com (X.L.); wangxn90@163.com (X.W.); jiangzj070517@163.com (Z.J.); xieyx0913@163.com (Y.X.); 2 Department of Science and Education, Huangshi Central Hospital, Affiliated Hospital of Hubei Polytechnic University, Huangshi 435000, China

**Keywords:** peripherally inserted central catheter, cancer patients, vein selection, temporal trends, retrospective study, PICC placement patterns

## Abstract

**Highlights:**

**What are the main findings?**

• The basilic vein was the dominant PICC insertion site from 2018 to 2023; however, the proportion of median cubital vein insertions increased substantially from 2024 and became the predominant site by 2025.
• Catheter insertion length and upper arm circumference exhibited statistically significant differences across age and tumor type groups; however, the effect sizes were generally small, indicating limited clinical relevance.

**What are the implications of the main findings?**

• The observed shift in venipuncture site selection reflects a notable change in real-world practice; however, as data on ultrasound utilization, operator experience, and protocol changes were not collected, the underlying causes remain speculative and warrant further investigation in future studies.
• As complication and clinical outcome data were not collected, these descriptive findings do not directly guide nursing protocols or resource allocation; prospective studies incorporating these variables are needed to evaluate the clinical impact of these observations.

**Abstract:**

**Background:** We aim to describe PICC placement characteristics, patient demographics, and temporal trends in insertion site selection among cancer patients at a tertiary hospital, and to provide real-world evidence on PICC practice patterns. **Methods:** Data from 1760 cancer patients who underwent PICC placement between January 2018 and December 2025 were analyzed. Descriptive statistics were used to summarize demographic characteristics and catheter parameters. Between-group comparisons were performed using the Kruskal–Wallis H test and Dunn’s test (with Bonferroni correction). Effect sizes (ε^2^, Cohen’s r), 95% confidence intervals (bootstrap method), and the Cochran–Armitage trend test for annual proportions were calculated. A multivariable general linear model (GLM) was employed to identify independent factors associated with catheter insertion length. **Results:** Among the 1760 patients, the right basilic vein was the most frequently used site. During 2018–2023, the basilic vein predominated; however, from 2024, the proportion of median cubital vein insertions increased substantially and became predominant by 2025. The annual proportions of both veins changed significantly over the study period (Cochran–Armitage trend test, *p* < 0.001 for both). Digestive and respiratory malignancies were the most common tumor types. Catheter insertion length and upper arm circumference exhibited statistically significant differences across age and diagnostic groups, albeit with small effect sizes. In the multivariable analysis, after adjusting for venipuncture site and other covariates, the magnitude of catheter length differences between the respiratory/thoracic tumor group and other major tumor groups was attenuated, indicating that the choice of anatomical site partially confounded the initial unadjusted findings. **Conclusions:** During 2024–2025, PICC placement shifted from the basilic vein to the median cubital vein, reflecting a notable change in real-world practice. Although the subgroup differences in catheter length and arm circumference were statistically significant, the clinical significance was limited. These descriptive findings do not directly guide nursing protocols. Future studies need to incorporate data on operator experience, ultrasound utilization, and complications to elucidate the mechanisms and clinical impact of this transition.

## 1. Introduction

Peripherally inserted central catheters (PICCs) are a type of central vascular access device (CVAD) inserted via peripheral veins—most commonly the basilic, cephalic, and median cubital veins—with the catheter tip positioned in the central venous circulation (the lower third of the superior vena cava or the subclavian vein) [1,2]. PICCs are designed to provide a safer and more durable venous access route for a variety of therapies, and have undergone substantial evolution since their introduction in 1975 [3]. The advantages of PICCs include ease of placement, extended dwell time, reduced frequency of venipuncture, and minimal vascular irritation [2].

Since their introduction in China, PICCs have been widely used in clinical practice for long-term transfusion, chemotherapy, and parenteral nutrition [4,5]. PICCs are particularly important for vulnerable populations, including pediatric and elderly patients, patients undergoing surgery or cancer chemotherapy, and individuals requiring enteral nutrition or medication delivery [6,7,8].

With the widespread use of PICCs, catheter maintenance during treatment-free intervals has emerged as a key concern. If PICC maintenance is delayed or inadequate, patients are at risk of PICC-related complications, including venous thrombosis, catheter-related bloodstream infections (CRBSIs), and mechanical complications (catheter dislodgement and occlusion) [9,10]. In China, PICCs are maintained by nurses; therefore, nurses’ knowledge and skills in PICC maintenance directly affect the quality of care, clinical outcomes, and patient safety [11,12,13].

Malignant tumors have become a major global public health challenge that seriously threatens human health and social development. According to GLOBOCAN 2024 data, an estimated 20.6 million new cancer cases and 9.8 million cancer-related deaths occurred worldwide, with both incidence and mortality rates remaining persistently high [14]. In 2024, China reported approximately 5.15 million new cancer cases and 2.58 million cancer-related deaths, underscoring a continued substantial cancer burden [15]. Premature mortality and years of life lost attributable to cancer globally have significantly impeded gains in life expectancy [16,17]. The accelerating pace of population aging has further compounded the cancer disease burden, exacerbating this constraint [18,19].

PICCs play an irreplaceable role in the comprehensive management of cancer. PICCs are essential for the administration of chemotherapeutic agents, which may be highly irritating to smaller peripheral veins; by contrast, PICCs extend to the larger central veins near the heart, offering greater durability and a lower risk of venous irritation or injury [20,21]. Beyond chemotherapy, cancer patients may require multiple additional therapies, including antibiotics, analgesics, antiemetics, and even total parenteral nutrition (TPN) [22]. PICCs enable the direct intravenous delivery of these agents and nutrients, ensuring rapid and efficient absorption, thereby substantially improving the long-term quality of life of cancer patients [23].

Huangshi is an important industrial city in China, characterized as a typical old industrial base with metallurgy, building materials, and equipment manufacturing as its dominant industries. Environmental pollution, occupational exposure, and other factors associated with long-term industrial development and urbanization may affect the incidence and mortality of malignant tumors among local residents; however, no substantial evidence is currently available. Concurrently, rapid socioeconomic development, lifestyle transitions, and continuous improvements in healthcare conditions have heightened the importance of PICC maintenance in enhancing the quality of nursing care.

Based on retrospective data from 1760 cancer patients with PICCs at a tertiary Grade A hospital in Huangshi between January 2018 and December 2025, this study aimed to characterize PICC placement patterns and temporal trends in insertion site selection, filling the gap in real-world descriptive evidence from tertiary hospitals in China.

## 2. Materials and Methods

### 2.1. Case Information

#### 2.1.1. Participants

A total of 1788 cancer patients who underwent PICC placement at a tertiary Grade A hospital in Huangshi, China, between January 2018 and December 2025 were initially enrolled. All patients underwent catheter placement only once during the study period; no repeat placements occurred. Exclusion criteria were as follows: (1) missing data on upper arm circumference (n = 22); (2) missing data on catheter insertion length (n = 6). Ultimately, 1760 patients were included in the final analysis.

This study was a retrospective medical record analysis, and all data were de-identified. The study protocol was approved by the Bioethical Safety Committee of Hubei Polytechnic University (approval number: yxy-20250109).

#### 2.1.2. Data Collection

The following data were retrospectively collected from the hospital electronic medical record system and the PICC registration system: (1) general demographic information, including age and sex; (2) catheter-related parameters, including date of catheterization, venipuncture site (basilic vein/median cubital vein/brachial vein/cephalic vein/axillary vein), catheter insertion length (cm), external catheter length (cm), and upper arm circumference (cm); and (3) clinical diagnostic information, including the classification of malignant tumor diagnoses.

### 2.2. Data Screening

Inclusion criteria were as follows: (1) a confirmed history of tumor diagnosis; (2) catheter placement performed at the hospital between 1 January 2018 and 31 December 2025; (3) complete data on age, sex, catheter placement parameters, and tumor diagnosis. Exclusion criteria were as follows: (1) missing data on upper arm circumference (n = 22); (2) missing data on catheter insertion length (n = 6).

An initial total of 1788 patients were identified; patients meeting the eligibility criteria were included in the statistical analysis, yielding a final study sample of 1760 patients.

### 2.3. Age Grouping

Age grouping was based on the age classification criteria of the International Statistical Classification of Diseases and Related Health Problems, 10th Revision (ICD-10), in conjunction with the demographic age structure of the Chinese population. Patients were categorized into three groups: ≤44 years, 45–64 years, and ≥65 years. The 45–64-year group was defined as middle-aged, consistent with the China Health Statistics Yearbook 2024, which classifies individuals aged ≥ 65 years as older adults.

### 2.4. Classification Criteria for Malignant Tumor Diagnoses

Tumor diagnoses were classified according to the diagnostic information recorded in the hospital medical record system. Malignant tumors were primarily categorized according to the anatomical system or primary site. Lymphomas and other hematological malignancies were classified under “Malignant neoplasms, stated or presumed to be primary, of lymphoid, hematopoietic and related tissue,” regardless of the anatomical site involved. Diagnostic categories with the same primary site and diagnostic type were consolidated to avoid redundant classification. The detailed distribution of malignant tumor types is presented in Supplementary Table S1.

### 2.5. Statistical Analysis

Data management and statistical analysis were performed using R software (version 4.4.1) and SPSS software (version 27.0). Figures were generated using Origin (version 2025) and the CNSknowall platform (https://cnsknowall.com/index.html#/HomePage, (accessed on 1 May 2026)). The normality of continuous variables was assessed using the Shapiro–Wilk test, Q–Q plots, and histograms. As the data did not follow a normal distribution, non-parametric tests were employed. Continuous variables are presented as medians (Q1, Q3), and categorical variables as frequencies and percentages. Temporal trends in the annual proportions of different PICC insertion sites were evaluated using the Cochran–Armitage trend test. Between-group comparisons were performed using the Kruskal–Wallis H test, followed by Dunn’s post hoc test with Bonferroni correction for multiple comparisons. To quantify the magnitude of differences, effect sizes were calculated: epsilon-squared (ε^2^) for the Kruskal–Wallis test and Cohen’s r (r = Z/√N) for Dunn’s pairwise comparisons. Additionally, 95% confidence intervals (CIs) for the median differences between groups were estimated using the bootstrap resampling method (2000 iterations). To control for potential confounding bias and to evaluate the independent effects of each variable on catheter insertion length, a multivariable general linear model (GLM) was constructed. Catheter insertion length was entered as the dependent variable, with sex, age group, side of placement, venipuncture site, tumor type, and year of placement included as fixed factors; results are expressed as unstandardized regression coefficients (B) with 95% CIs. All statistical tests were two-sided, and *p* < 0.05 was considered statistically significant. The R packages used included dplyr, readxl, openxlsx, FSA, rcompanion, and boot.

## 3. Results

### 3.1. Baseline Characteristics of Cancer Patients with PICCs

**Figure 1:**
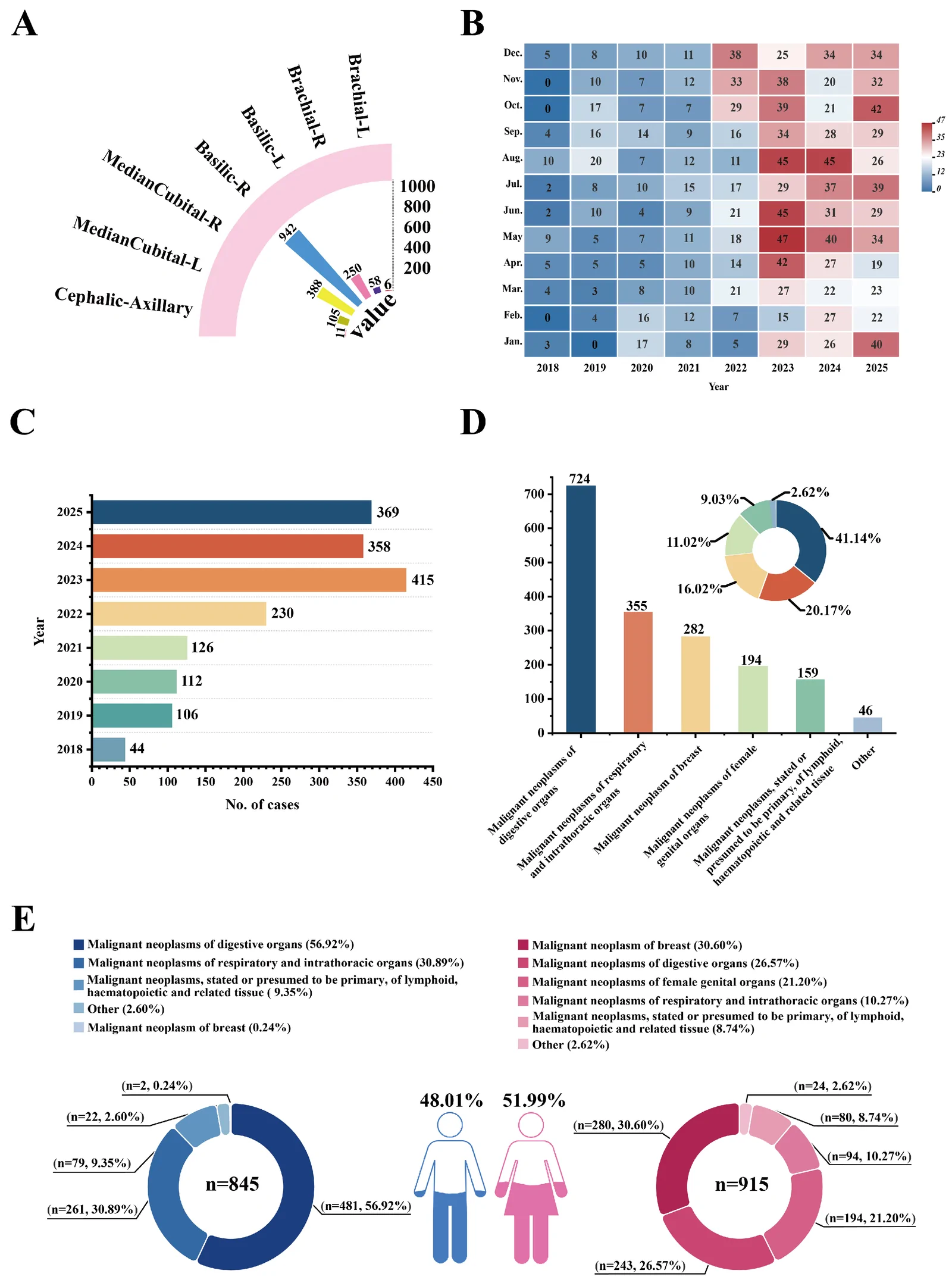
Baseline characteristics of cancer patients with PICCs. (**A**) Distribution of catheter insertion sites. (**B**) Heatmap of monthly distribution of catheter placements (2018–2025). (**C**) Annual distribution of total catheter placements. (**D**) Overall composition of malignant tumor types among PICC patients. (**E**) Composition of malignant tumor types among PICC patients stratified by sex.

A total of 1760 cancer patients who underwent PICC placement were included in this study. Of these, 845 (48.01%) were male and 915 (51.99%) were female. The PICC insertion sites, annual and monthly distribution of catheter placements, overall composition of malignant tumors, and tumor composition stratified by sex are presented in Figure 1.

#### 3.1.1. PICC Insertion Vein Sites

The most commonly used insertion vein was the right basilic vein (Basilic-R) (n = 942), followed by the right median cubital vein (Median Cubital-R) (n = 388). The left basilic vein (Basilic-L) and left median cubital vein (Median Cubital-L) were used in 250 and 105 cases, respectively. The cephalic and axillary veins (Cephalic–Axillary) were rarely used (n = 11). Among brachial vein insertions, the right brachial vein (Brachial-R) was used in 58 cases, whereas the left brachial vein (Brachial-L) was used in six cases (n = 6) (Figure 1A).

#### 3.1.2. Temporal Distribution of PICC Placements

The annual and monthly distributions of PICC placements from 2018 to 2025 are shown in Figure 1B,C. The monthly number of PICC placements exhibited an overall increasing trend over time, with some fluctuation across months within each year (Figure 1B). The total number of catheter placements was lowest in 2018 (n = 44), increased markedly after 2022, and peaked in 2023 (n = 415). Case numbers in 2024 and 2025 remained at high levels, at 358 and 369, respectively (Figure 1C). As absolute case counts are subject to fluctuations in the total annual number of catheter placements, we calculated the annual proportions of each insertion site; the results are presented in Table 1.

From 2018 to 2023, the proportion of basilic vein PICC insertions rose from 63.64% in 2018 to approximately 91% or higher during 2019–2023. Their proportion decreased markedly from 92.05% in 2023 to 62.01% in 2024, and further dropped to 6.23% in 2025. By contrast, the proportion of median cubital vein insertions was only 0.24% in 2023, and increased rapidly to 34.08% in 2024, reaching 93.50% in 2025, which made this site the most commonly selected vessel for PICC placement in 2025.

Cochran–Armitage trend tests demonstrated significant overall temporal changes in the annual proportional distribution for both the basilic vein ($\chi_{trend}^{2}$ = 441.11, *p* < 0.001) and median cubital vein ($\chi_{trend}^{2}$ = 521.29, *p* < 0.001). The utilization proportion of the brachial vein fluctuated across the study period and peaked at 7.71% in 2023, with no statistically significant overall temporal change observed ($\chi_{trend}^{2}$ = 0.52, *p* = 0.471). For the cephalic and axillary veins, the annual case numbers were too small to support a valid Cochran–Armitage trend test. Therefore, trend testing was not performed for these two veins, and their data are presented descriptively only.

#### 3.1.3. Distribution of Malignant Tumor Types Among PICC Patients

Among the malignant tumor types, malignant neoplasms of digestive organs were the most prevalent (n = 724; 41.14%), followed by malignant neoplasms of respiratory and intrathoracic organs (n = 355; 20.17%), malignant neoplasms of the breast (n = 282; 16.02%), malignant neoplasms of female genital organs (n = 194; 11.02%), and malignant neoplasms, stated or presumed to be primary, of lymphoid, hematopoietic and related tissue (n = 159; 9.03%). Malignancies of other sites accounted for 46 cases (n = 46; 2.62%) (Figure 1D).

#### 3.1.4. Sex Distribution and Malignant Tumor Composition Among PICC Patients

Male patients accounted for 845 cases (48.01%) and female patients for 915 cases (51.99%). Among male patients, malignant neoplasms of digestive organs were the most common (n = 481; 56.92%), followed by malignant neoplasms of respiratory and intrathoracic organs (n = 261; 30.89%), malignant neoplasms, stated or presumed to be primary, of lymphoid, hematopoietic and related tissue (n = 79; 9.35%), malignancies of other sites (n = 22; 2.60%), and malignant neoplasms of the breast (n = 2; 0.24%). Among female patients, malignant neoplasms of the breast were the most common (n = 280; 30.60%), followed by malignant neoplasms of digestive organs (n = 243; 26.57%), malignant neoplasms of female genital organs (n = 194; 21.20%), malignant neoplasms of respiratory and intrathoracic organs (n = 94; 10.27%), malignant neoplasms, stated or presumed to be primary, of lymphoid, hematopoietic and related tissue (n = 80; 8.74%), and malignancies of other sites (n = 24; 2.62%) (Figure 1E).

### 3.2. Age and Tumor Type Distribution Among Patients with PICCs

**Figure 2:**
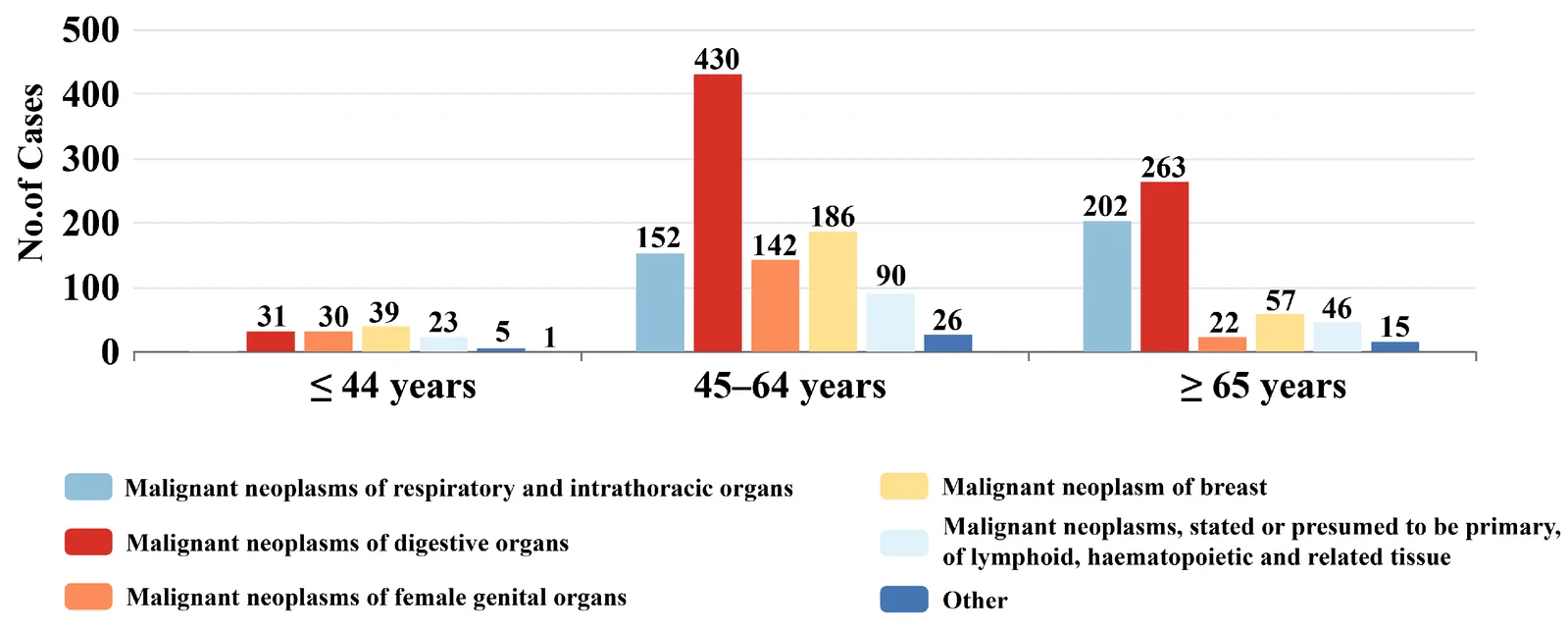
Distribution of malignant tumor types across age groups among PICC patients.

Patients were categorized into three age groups: ≤44 years, 45–64 years, and ≥65 years. The case counts and proportional distributions of malignant tumor types across age groups are presented in Figure 2.

The ≤44-year group had the fewest cases overall, comprising 39 malignant neoplasms of the breast (30.23%), 31 malignant neoplasms of digestive organs (24.03%), 30 malignant neoplasms of female genital organs (23.25%), 23 malignant neoplasms, stated or presumed to be primary, of lymphoid, hematopoietic and related tissue (17.83%), five malignancies of other sites (3.88%), and one malignant neoplasm of respiratory and intrathoracic organs (0.78%).

The 45–64-year group had the largest number of cases, comprising 430 malignant neoplasms of digestive organs (41.91%), 186 malignant neoplasms of the breast (18.14%), 152 malignant neoplasms of respiratory and intrathoracic organs (14.81%), 142 malignant neoplasms of female genital organs (13.84%), 90 malignant neoplasms, stated or presumed to be primary, of lymphoid, hematopoietic and related tissue (8.77%), and 26 malignancies of other sites (2.53%). Malignant neoplasms of digestive organs were the most prevalent tumor type in this age group.

The ≥65-year group comprised 263 malignant neoplasms of digestive organs (43.47%), 202 malignant neoplasms of respiratory and intrathoracic organs (33.39%), 57 malignant neoplasms of the breast (9.42%), 46 malignant neoplasms, stated or presumed to be primary, of lymphoid, hematopoietic and related tissue (7.60%), 22 malignant neoplasms of female genital organs (3.64%), and 15 malignancies of other sites (2.48%). Malignant neoplasms of digestive organs/respiratory and intrathoracic organs together constituted the principal tumor types in the older adult population (Figure 2).

### 3.3. Distribution of PICC Placements Among Cancer Patients

#### 3.3.1. Distribution of Catheter Parameters by Venipuncture Site

**Figure 3:**
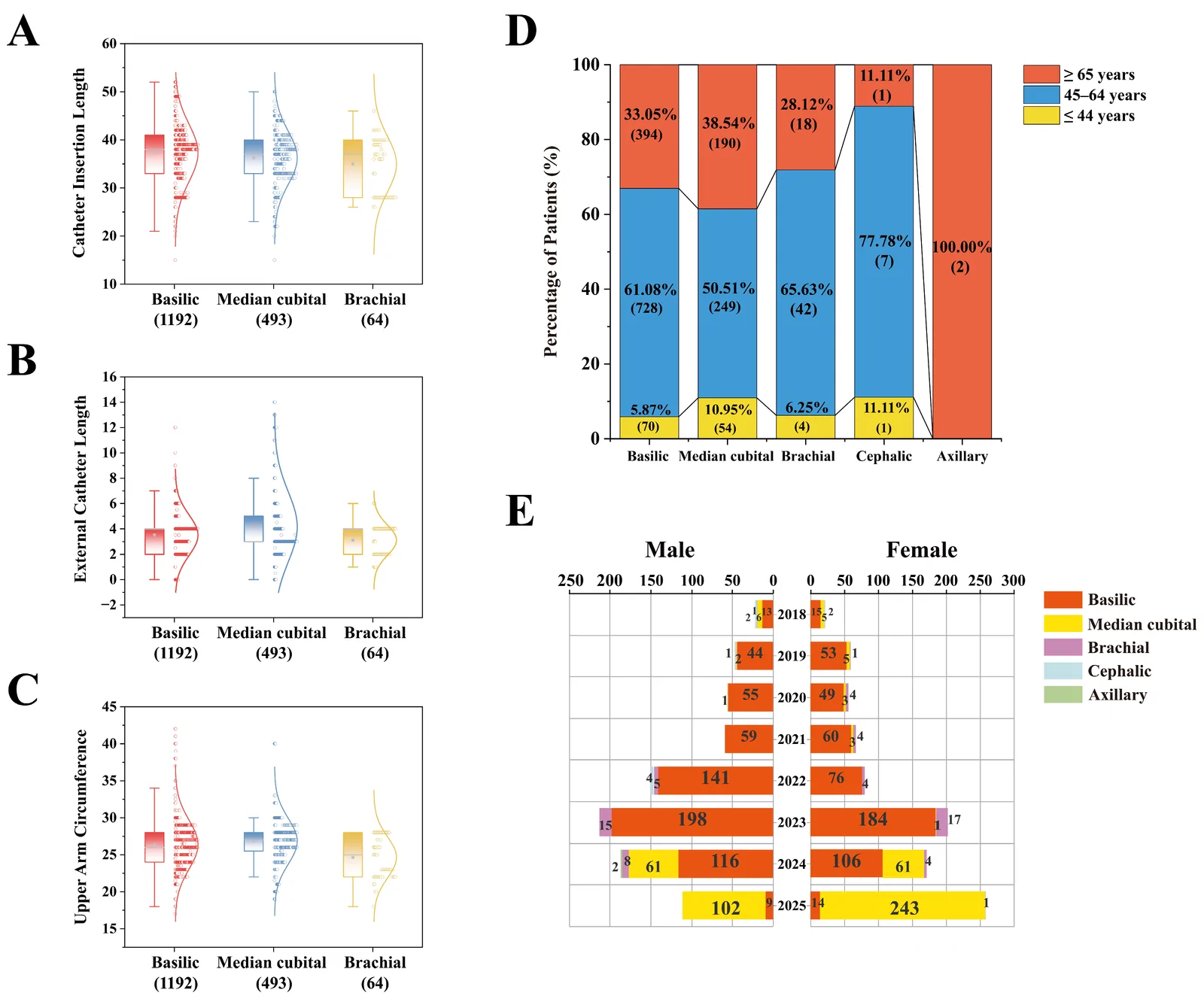
Catheter parameters and patient characteristics by venipuncture site. (**A**) Distribution of in vivo catheter insertion length by insertion site. (**B**) Distribution of external catheter length by insertion site. (**C**) Distribution of upper arm circumference by insertion site. In subpanels (**A**–**C**), data are displayed as half-box plots combined with scatter plots and normal distribution curves. The outer curve illustrates the normal probability density distribution of the data. The solid line within the box represents the median, and the square marker represents the mean. The lower and upper boundaries of the box correspond to the first (Q1) and third (Q3) quartiles, respectively. The whiskers extend to the extreme values within 1.5 times the interquartile range (IQR) of the box. The adjacent scatter points represent raw data points. The sample size (n) for each group is indicated on the *x*-axis in brackets. (**D**) Age distribution of patients across different venipuncture sites. (**E**) Annual distribution trends of insertion sites stratified by sex.

The distribution of catheter parameters across different venipuncture sites is shown in Figure 3. This study primarily analyzed three main venipuncture veins: the basilic vein (n = 1192), the median cubital vein (n = 493), and the brachial vein (n = 64). For catheter insertion length, the median for the basilic vein was 38.0 cm (Q1–Q3: 33.0–41.0; range: 15.0–52.0), for the median cubital vein was 37.0 cm (Q1–Q3: 33.0–40.0; range: 15.0–50.0), and for the brachial vein was 37.0 cm (Q1–Q3: 28.0–40.0; range: 26.0–46.0) (Figure 3A).

For external catheter length, the median for the basilic vein was 4.0 cm (Q1–Q3: 2.0–4.0; range: 0.0–12.0), for the median cubital vein was 3.0 cm (Q1–Q3: 3.0–5.0; range: 0.0–14.0), and for the brachial vein was 4.0 cm (Q1–Q3: 2.0–4.0; range: 1.0–6.0) (Figure 3B).

For upper arm circumference, the median for patients with basilic vein placement was 26.0 cm (Q1–Q3: 24.0–28.0; range: 17.0–42.0), for patients with median cubital vein placement was 27.0 cm (Q1–Q3: 25.5–28.0; range: 19.0–40.0), and for patients with brachial vein placement was 25.0 cm (Q1–Q3: 22.0–28.0; range: 18.0–28.0) (Figure 3C).

#### 3.3.2. Age Distribution by Insertion Vein

The age composition of patients across PICC insertion veins is shown in Figure 3D. Overall, the 45–64-year age group constituted the largest proportion, followed by the ≥65-year group, while the ≤44-year group was relatively underrepresented. Among patients with basilic vein insertions, 728 (61.08%) were aged 45–64 years, 394 (33.05%) were aged ≥ 65 years, and 70 (5.87%) were aged ≤ 44 years. Among patients with median cubital vein insertions, 249 (50.51%) were aged 45–64 years, 190 (38.54%) were aged ≥ 65 years, and 54 (10.95%) were aged ≤ 44 years. Among patients with brachial vein insertions, 42 (65.63%) were aged 45–64 years, 18 (28.12%) were aged ≥ 65 years, and four (6.25%) were aged ≤ 44 years. Case counts for cephalic and axillary vein insertions were limited: among cephalic vein insertions, seven cases were in the 45–64-year age group, with one case each in the ≤ 44-year and ≥65-year groups; both axillary vein insertions occurred in patients aged ≥ 65 years (n = 2).

#### 3.3.3. Temporal Trends in Insertion Vein Selection by Sex

Trends in insertion vein selection by sex from 2018 to 2025 are shown in Figure 3E. Overall, the trends in vein selection were largely consistent between male and female patients. From 2018 to 2023, the basilic vein was the predominant insertion site, with 198 male and 184 female patients receiving basilic vein insertions in 2023. Beginning in 2024, the pattern of vein selection began to shift: among male patients, 116 received basilic vein insertions and 61 received median cubital vein insertions; among female patients, 106 received basilic vein insertions and 61 received median cubital vein insertions. By 2025, the median cubital vein had become the predominant insertion site, with 102 male patients receiving median cubital vein insertions compared with nine via the basilic vein, and 243 female patients receiving median cubital vein insertions compared with 14 via the basilic vein. Other insertion veins (brachial, cephalic, and axillary) were used infrequently, with minimal annual variation.

### 3.4. PICC Parameters by Age Group and Tumor Classification

#### 3.4.1. Comparison of Catheter Parameters Across Age Groups

The distribution of catheter parameters across age groups is presented in Table 2. The Kruskal–Wallis H test revealed statistically significant differences among the three groups for catheter insertion length (H = 21.49, *p* < 0.001) and upper arm circumference (H = 11.30, *p* = 0.004), whereas external catheter length did not differ significantly across age groups (H = 1.69, *p* = 0.429).

Pairwise comparisons were performed using Dunn’s test with Bonferroni correction, and the results are presented in Table 3. For catheter insertion length, the ≤44-year group had significantly greater lengths than both the 45–64-year group (median difference: 1.00 cm, 95% CI: 1.00–3.00; Z = 4.64, adjusted *p* < 0.001; r = 0.111) and the ≥65-year group (median difference: 1.00 cm, 95% CI: 1.00–2.00; Z = 3.95, adjusted *p* < 0.001; r = 0.095). However, all effect sizes were small (r < 0.12). For upper arm circumference, the ≥65-year group was slightly larger than the 45–64-year group (median difference: −1.00 cm, 95% CI: −1.00–0.00; Z = 3.35, adjusted *p* = 0.002; r = 0.080); all remaining comparisons were not statistically significant (all adjusted *p* > 0.05). All effect sizes for upper arm circumference were negligible (|r| < 0.10). As the omnibus test for external catheter length was not statistically significant, pairwise comparisons were not performed.

#### 3.4.2. Comparison of Catheter Parameters Across Tumor Classifications

The distribution of catheter-related parameters across diagnostic groups is presented in Table 4. The Kruskal–Wallis H test revealed statistically significant differences among groups for catheter insertion length (H = 134.17, *p* < 0.001), upper arm circumference (H = 15.42, *p* = 0.009), and external catheter length (H = 14.25, *p* = 0.014).

For each parameter, all pairwise comparisons were performed using Dunn’s test with Bonferroni correction. To avoid selective reporting, the complete results (including all comparisons without statistical significance) are provided in Table S2A (upper arm circumference), S2B (external catheter length), and S2C (catheter insertion length), accompanied by Z values, adjusted *p* values, median differences (95% CIs), and effect sizes (r).

Regarding catheter insertion length, the malignant neoplasms of respiratory and intrathoracic organs group had a significantly shorter length than the malignant neoplasms of digestive organs group (median difference: −5.00 cm, 95% CI: −6.00 to −3.00; Z = −9.63, adjusted *p* < 0.001; r = −0.229), the malignant neoplasms of the breast group (median difference: −6.00 cm, 95% CI: −6.00 to −4.00; Z = −8.51, adjusted *p* < 0.001; r = −0.203), and the malignant neoplasms, stated or presumed to be primary, of lymphoid, hematopoietic and related tissue group (median difference: −6.00 cm, 95% CI: −6.00 to −4.00; Z = −8.10, adjusted *p* < 0.001; r = −0.193). The malignant neoplasms of female genital organs group also differed significantly from both the malignant neoplasms of the breast group (median difference: −2.00 cm, 95% CI: −3.00 to −1.00; Z = −4.78, adjusted *p* < 0.001; r = −0.114) and the digestive group (median difference: −1.00 cm, 95% CI: −2.00 to −1.00; Z = −4.83, adjusted *p* < 0.0.01; r = −0.115). Furthermore, the malignant neoplasms, stated or presumed to be primary, of lymphoid, hematopoietic and related tissue group differed significantly from the malignant neoplasms of female genital organs group (median difference: 2.00 cm, 95% CI: 1.50 to 3.00; Z = 5.04, adjusted *p* < 0.001; r = 0.120). All remaining comparisons were not statistically significant.

For upper arm circumference, only the difference between the malignant neoplasms of respiratory and intrathoracic organs group and the malignant neoplasms of the breast group was statistically significant (Z = −3.24, adjusted *p* = 0.018; median difference: 0.00 cm, 95% CI: −1.00 to 0.50; r = −0.077) (see Table S2A).

For external catheter length, only the difference between the malignant neoplasms of respiratory and intrathoracic organs group and the malignant neoplasms of digestive organs group was statistically significant (Z = −2.95, adjusted *p* = 0.048; median difference: −1.00 cm, 95% CI: −1.00 to −1.00; r = −0.070) (see Table S2B).

Notably, all effect sizes for upper arm circumference and external catheter length were negligible (|r| < 0.10), and the median differences across all comparisons were minimal (0 to −1 cm), suggesting that these statistical differences were primarily driven by the large sample size and have limited clinical practical significance. For catheter insertion length, the effect sizes involving the malignant neoplasms of respiratory and intrathoracic organs group reached the small effect threshold (|r| = 0.193 to 0.229), with absolute median differences of 5 to 6 cm, which may be of more clear clinical interest.

#### 3.4.3. Multivariable Analysis of Catheter Insertion Length

To further evaluate the independent effects of each variable on catheter insertion length, a multivariable general linear model (GLM) was constructed, with sex, age group, side of placement, venipuncture site, tumor type, and year of placement included as fixed factors. The results (Table 5) showed that, after adjusting for other covariates, side of placement, venipuncture site, and certain tumor types remained independently associated with catheter insertion length. Specifically, left-sided placement had significantly greater lengths than right-sided placement (B = 1.01, 95% CI: 0.43–1.59, *p* < 0.001). Compared with the brachial vein, catheter insertion length was significantly longer via the basilic vein (B = 2.69, 95% CI: 1.26–4.12, *p* < 0.001) and the median cubital vein (B = 1.33, 95% CI: 0.13–2.53, *p* = 0.030); furthermore, patients with lymphoid/hematopoietic tumors, breast tumors, female genital organ tumors, and digestive organ tumors all exhibited longer catheter insertion lengths than the “other” tumor group (all *p* < 0.05). Notably, although the unadjusted univariate analysis initially revealed that the respiratory/thoracic tumor group had significantly shorter catheter lengths (e.g., compared with the digestive organ group), in the fully adjusted model, there was no significant difference between this group and the reference category (“other”) (B = −0.13, 95% CI: −1.63 to 1.38, *p* = 0.870). As this adjusted *p* value reflects only the comparison with the reference group—rather than direct adjusted pairwise comparisons between major tumor types—it cannot statistically confirm that the original between-group differences have fully disappeared. Nonetheless, this finding indicates that the initially observed differences were at least partially confounded by factors such as anatomical site selection.

## 4. Discussion

### 4.1. Composition of Malignant Tumor Types and Demographic Characteristics Among Patients with PICCs

The malignant tumor spectrum in this study was dominated by malignant neoplasms of digestive organs, followed by malignant neoplasms of respiratory and intrathoracic organs and malignant neoplasms of the breast.

It should be explicitly noted that the distribution characteristics reflect only the tumor type composition of a specific subgroup of patients who met the indications for long-term intravenous therapy and underwent PICC placement at a single center; these findings cannot directly represent the overall disease burden of malignant tumors in the general population of the Huangshi region. This discrepancy fundamentally arises from the inherent heterogeneity in the clinical need for long-term venous access across different tumor types.

Global cancer statistics from 2024 identified lung cancer, female breast cancer, colorectal cancer, prostate cancer, and gastric cancer as the top five newly diagnosed malignancies [14]. According to 2024 cancer epidemiological data for China, lung cancer, thyroid cancer, colorectal cancer, breast cancer, and liver cancer ranked highest in incidence [15].

Sex-stratified analysis in the present study further demonstrated notable sex-based differences in the tumor spectrum: neoplasms of the breast accounted for 30.71% of cases among female patients, whereas malignant neoplasms of digestive organs and malignant neoplasms of respiratory and intrathoracic organs were more prevalent among male patients. The age distribution in this study was concentrated in the 45–64-year range, closely aligning with the peak incidence ages reported in national cancer registry data (60–64 years for males and 50–54 years for females) [15].

This finding suggests that, against the backdrop of continuing population aging in China, the demand for long-term intravenous therapy—of which PICCs are a core venous access option for patients requiring extended chemotherapy and infusion—may continue to rise. However, our study did not collect data on workload, resource use, or staffing, and therefore cannot directly inform resource allocation. Future research incorporating these variables is needed to evaluate the broader implications of these demographic trends for PICC services.

### 4.2. Changes in Insertion Vein Selection

Owing to its anatomical advantages—including a larger diameter, fewer venous valves, and a straighter course—the basilic vein is recommended by the Infusion Nurses Society (INS) guidelines as the preferred vein for PICC placement [24]. In this study, the utilization rate of the basilic vein for PICC insertion from 2018 to 2023 far exceeded that of other veins. However, a shift emerged in 2024, with the utilization rate of the median cubital vein increasing markedly by 2025.

However, in the absence of data on catheter dwell outcomes, complication records, clear anatomical definitions of insertion sites, protocol changes, and operator-related variables, only exploratory hypotheses are proposed: we hypothesize that departmental-level updates to procedural protocols may have contributed to this shift. Another possibility is that the median cubital vein’s superficial location and ease of hemostatic compression may offer safety advantages in selected patients, such as those with coagulopathy. These explanations remain speculative and should be regarded as hypotheses rather than data-supported mechanisms.

### 4.3. Clinical Interpretation of Catheter Parameter Differences

In our study, catheter insertion length differed significantly across age groups in the univariable analysis (H = 21.49, *p* < 0.001); the effect size was small, indicating limited clinical relevance. The ≤44-year group had a median catheter insertion length of 39.0 cm, which was longer than that of both the 45–64-year group (38.0 cm) and the ≥65-year group (38.0 cm), with pairwise differences reaching statistical significance (adjusted *p* < 0.001 for both comparisons); however, the effect sizes remained small. This difference may be related to the correspondingly longer anatomical distance from the venipuncture site to the mid-to-lower segment of the superior vena cava.

Other studies have found that age also exerts an independent effect on catheter insertion length during right-arm placement procedures [25]. A separate study based on anteroposterior chest radiograph measurements further validated the correlation between imaging-derived anatomical parameters and catheter length [26]. Although previous studies in neonatal populations have suggested that individualized catheterization protocols may reduce tip malposition and associated complications, our study did not assess final tip position, patient height, or complications. Therefore, we cannot conclude that individualized catheter placement is applicable across age groups. These findings remain descriptive and do not provide evidence to support clinical recommendations for individualized catheterization strategies.

### 4.4. Exploratory Findings on Inter-Group Differences in Catheter Insertion Length

Catheter insertion length differed significantly across diagnostic groups (H = 134.17, *p* < 0.001). The median catheter insertion length in the malignant neoplasms of respiratory and intrathoracic organs group was only 33.0 cm, which was significantly shorter than that in the malignant neoplasms of digestive organs group (38.0 cm), the malignant neoplasms, stated or presumed to be primary, of lymphoid, hematopoietic and related tissue group (39.0 cm), and the malignant neoplasms of the breast group (39.0 cm) (adjusted *p* < 0.001 for all pairwise comparisons). This difference warrants particular attention: the optimal position of the PICC tip should be located in the lower third of the superior vena cava to the cavoatrial junction [24]. Previous studies have confirmed that catheter tip position is an independent risk factor for PICC-related venous thrombosis [27]; furthermore, suitable catheter insertion length has been identified as a protective factor against unplanned catheter removal [28].

However, it should be noted that the study lacks data on the actual final tip position, patient height, anatomy, complications, or clinical outcomes. A shorter length does not necessarily imply inadequate placement, as the required length depends on various individual characteristics and the puncture site.

The specific reason for the shorter catheter insertion length observed in patients with malignant neoplasms of respiratory and intrathoracic organs remains unclear; whether this is related to preferences in venipuncture site selection (e.g., a tendency to choose veins closer to the heart) or to patient positioning during the procedure requires further investigation.

### 4.5. Interpretation of Upper Arm Circumference and External Catheter Length

This study found significant differences in upper arm circumference across diagnostic groups; however, post hoc pairwise comparisons revealed a significant difference only between the malignant neoplasms of respiratory and intrathoracic organs group and the malignant neoplasms of the breast group. However, given that a significant difference was observed only between a single pair of groups and the clinical magnitude of the difference was small, the clinical significance of this finding should be interpreted with caution.

External catheter length showed a marginally significant difference across diagnostic groups, with a significant pairwise difference observed only between the malignant neoplasms of respiratory and intrathoracic organs group and the malignant neoplasms of digestive organs group. However, the majority of pairwise differences were non-significant, with median differences of approximately 1 cm and small effect sizes. The clinical significance of external catheter length lies primarily in its association with catheter fixation stability; however, the magnitude of the differences observed in this study is insufficient to constitute clear evidence supporting the adjustment of external catheter length based on diagnostic category.

In summary, the differences in upper arm circumference and external catheter length across diagnostic groups were small, with all effect sizes being negligible (|r| < 0.10). These findings indicate that these two parameters are highly consistent across tumor types, supporting the use of universal catheterization protocols for the majority of cancer patients. Because complications and clinical outcomes were not assessed, the clinical relevance of these findings remains uncertain; they are descriptive only and do not support adjustments to catheterization protocols based on tumor type.

### 4.6. Limitations of This Study

(1) Single-center data. Data completeness was limited by reliance on the electronic medical record system and the PICC registration system, which may be subject to misreporting and underreporting.
(2) Selection bias. The study population comprised only a subset of patients with malignant tumors who underwent PICC placement in Huangshi. Because this study was based exclusively on populations recorded in a single hospital’s medical registration system, the actual number of patients receiving PICCs may have been underestimated.
(3) Geographic limitations. The data were derived from a single region, whose demographic composition and healthcare accessibility may differ from those of other regions in China. Moreover, hospitals across different regions and tiers may have markedly different catheterization practices; therefore, extrapolation of these findings should be undertaken with caution.
(4) Data collection constraints. Specifically, this study lacked key clinical parameters such as patient height measurements and radiographic or intracavitary electrocardiographic (ECG) confirmation of catheter tip position. Moreover, follow-up data on post-catheterization complications (e.g., catheter-related bloodstream infections, thrombosis, and phlebitis) were not available, precluding assessment of the true impact of changes in vein selection on patient outcomes. Key procedural variables such as operator experience and ultrasound equipment model were not collected, thereby weakening the explanatory power regarding the reasons for the 2025 transition.
(5) Clinical significance. The differences in upper arm circumference and external catheter length between diagnostic groups were significant only in select pairwise comparisons, with small clinical magnitudes; the clinical significance of these findings should, therefore, be interpreted cautiously.

In summary, this study described the insertion site characteristics, age distribution, and recent venous selection trends among 1760 patients with malignant tumors who underwent PICC placement at a tertiary Grade A hospital in Huangshi between 2018 and 2025. Given that the present study did not collect complication or clinical outcome data, the current results are insufficient to draw definitive conclusions regarding the appropriateness of catheter placement protocols, nor can they support any specific clinical recommendations. Accordingly, future studies incorporating patient individual characteristics, operator experience, and complication data may further investigate the potential driving factors behind the shift in venous selection. Specifically, future studies may evaluate the feasibility of routine post-procedural radiographic tip position verification and preliminarily explore the potential association between intracavitary electrocardiogram navigation and catheter length selection, as well as final tip position.

## 5. Conclusions

Based on retrospective clinical data from 1760 patients with malignant tumors who underwent PICC placement at a tertiary Grade A hospital in Huangshi between 2018 and 2025, this study found that the basilic vein was the predominant insertion site from 2018 to 2024, whereas the proportion of median cubital vein insertions increased substantially in 2025, establishing it as a major venipuncture site. Catheter insertion length and upper arm circumference exhibited statistically significant differences across age strata and tumor type groups; however, these differences were clinically small. These descriptive findings may inform future research but do not directly guide nursing protocols.

The included population was predominantly composed of patients with malignant neoplasms of digestive organs (41.14%) and malignant neoplasms of respiratory and intrathoracic organs (20.17%), with middle-aged and older adults representing the majority of PICC recipients. In summary, future studies should further incorporate variables such as operator experience, ultrasound equipment utilization, and PICC-related complications to explore the potential mechanisms underlying changes in catheterization characteristics and their associations with patient outcomes.

## Figures and Tables

**Table 1:** Annual distribution of PICC insertion sites from 2018 to 2025, n (%).

Year	Basilic Vein, n (%)	Median Cubital Vein, n (%)	Cephalic Vein, n (%)	Brachial Vein, n (%)	Axillary Vein, n (%)	Total, n
2018	28 (63.64%)	11 (25.00%)	4 (9.09%)	1 (2.27%)	0 (0.00%)	44
2019	97 (91.51%)	7 (6.60%)	1 (0.94%)	1 (0.94%)	0 (0.00%)	106
2020	104 (92.86%)	4 (3.57%)	0 (0.00%)	4 (3.57%)	0 (0.00%)	112
2021	119 (94.44%)	3 (2.38%)	0 (0.00%)	4 (3.17%)	0 (0.00%)	126
2022	217 (94.35%)	0 (0.00%)	4 (1.74%)	9 (3.91%)	0 (0.00%)	230
2023	382 (92.05%)	1 (0.24%)	0 (0.00%)	32 (7.71%)	0 (0.00%)	415
2024	222 (62.01%)	122 (34.08%)	0 (0.00%)	12 (3.35%)	2 (0.56%)	358
2025	23 (6.23%)	345 (93.50%)	0 (0.00%)	1 (0.27%)	0 (0.00%)	369
$\chi_{trend}^{2}$	441.11	521.29	-	0.52	-	
*p*-value	<0.001	<0.001	-	0.471	-	

Note: Percentages were calculated within each calendar-year total sample to reflect the proportional preference for each insertion vein, independent of yearly fluctuation in overall PICC procedure volume. Percentages are rounded to two decimal places; minor summation deviations may occur due to rounding.

**Table 2:** Comparison of catheter parameters among different age groups.

Age Group	N	Catheter Insertion Length (cm)	Upper Arm Circumference (cm)	External Catheter Length (cm)
≤44 years	129	39.0 (37.0, 41.0)	26.0 (25.0, 28.0)	3.0 (3.0, 4.0)
45–64 years	1026	38.0 (33.0, 40.0)	26.0 (24.0, 28.0)	4.0 (3.0, 4.0)
≥65 years	605	38.0 (33.0, 41.0)	27.0 (24.0, 28.0)	3.0 (2.0, 4.0)
Total	1760	38.0 (33.0, 40.0)	26.5 (24.0, 28.0)	3.0 (2.0, 4.0)
H/*p*-value		21.49/<0.001	11.30/0.004	1.69/0.429
ε^2^		0.012	0.006	0.001

Note: Data are presented as medians (Q1, Q3). Between-group comparisons were performed using the Kruskal–Wallis H test. Effect sizes are expressed as epsilon-squared (ε^2^). According to Cohen’s criteria, ε^2^ < 0.01 is considered negligible, 0.01 ≤ ε^2^ < 0.06 small, 0.06 ≤ ε^2^ < 0.14 medium, and ε^2^ ≥ 0.14 large.

**Table 3:** Pairwise comparisons of catheter insertion length and upper arm circumference among age groups (Dunn’s test with Bonferroni correction).

Variable	Comparison	Z	Adjusted *p*	Median Diff. (95% CI)	Effect Size (r)
Catheter insertion length					
	≤44 vs. 45–64 years	4.64	<0.001	1.00 (1.00, 3.00)	0.111
	≤44 vs. ≥65 years	3.95	<0.001	1.00 (1.00, 2.00)	0.095
	≥65 vs. 45–64 years	0.98	0.983	0.00 (−1.00, 0.00)	0.023
Upper arm circumference					
	≤44 vs. 45–64 years	0.95	1.000	1.00 (−1.00, 1.00)	0.023
	≤44 vs. ≥65 years	−0.84	1.000	0.00 (−1.00, 0.00)	−0.020
	≥65 vs. 45–64 years	3.35	0.002	−1.00 (−1.00, 0.00)	0.080

Note: Pairwise comparisons were performed using Dunn’s test with Bonferroni correction. The median difference was calculated as: median of the first group − median of the second group (i.e., consistent with the order of groups listed in the comparison column of the table). A positive value indicates that the first group had a larger value, and a negative value indicates that the first group had a smaller value. Effect sizes are expressed as Cohen’s r (r = Z/√N). According to Cohen’s criteria, |r| < 0.10 is considered negligible, 0.10 ≤ |r| < 0.30 small, 0.30 ≤ |r < 0.50 medium, and |r| ≥ 0.50 large.

**Table 4:** Comparison of catheter parameters across different tumor classification groups.

Diagnostic Group	N	Catheter Insertion Length (cm)	Upper Arm Circumference (cm)	External Catheter Length (cm)
Malignant neoplasms of digestive organs	724	38.0 (34.0, 42.0)	27.0 (24.0, 28.0)	4.0 (3.0, 4.0)
Malignant neoplasms of respiratory and intrathoracic organs	355	33.0 (28.0, 38.0)	26.0 (24.0, 28.0)	3.0 (2.0, 4.8)
Malignant neoplasms of the breast	282	39.0 (35.0, 41.0)	26.0 (25.0, 28.0)	3.0 (3.0, 4.0)
Malignant neoplasms of female genital organs	194	37.0 (33.0, 39.0)	26.0 (24.0, 28.0)	3.0 (3.0, 4.0)
Malignant neoplasms, stated or presumed to be primary, of lymphoid, hematopoietic and related tissue	159	39.0 (37.0, 40.0)	27.0 (25.0, 28.0)	3.0 (3.0, 4.0)
Other	46	37.0 (33.0, 39.0)	27.0 (24.0, 28.0)	3.0 (2.2, 4.0)
Total	1760	38.0 (33.0, 40.0)	26.5 (24.0, 28.0)	3.0 (2.0, 4.0)
H/*p*-value		134.17/<0.001	15.42/0.009	14.25/0.014
ε^2^		0.076	0.009	0.008

Note: Data are presented as medians (Q1, Q3). Between-group comparisons were performed using the Kruskal–Wallis H test. Effect sizes are expressed as epsilon-squared (ε^2^). According to Cohen’s criteria, ε^2^ < 0.01 is considered negligible, 0.01 ≤ ε^2^ < 0.06 small, 0.06 ≤ ε^2^ < 0.14 medium, and ε^2^ ≥ 0.14 large.

**Table 5:** Multivariable general linear model analysis of factors associated with catheter insertion length.

Variables	B	95% CI	*p*-Value
Sex			
Female	Reference	-	-
Male	0.49	−0.07, 1.05	0.088
Age group			
45–64 years	Reference	-	-
≤44 years	0.58	−0.31, 1.48	0.203
≥65 years	0.42	−0.09, 0.93	0.105
Insertion side			
Right	Reference	-	-
Left	1.01	0.43, 1.59	<0.001
Venipuncture site			
Brachial vein	Reference	-	-
Basilic vein	2.69	1.26, 4.12	<0.001
Median cubital vein	1.33	0.13, 2.53	0.030
Tumor classification			
Other	Reference	-	-
Malignant neoplasms of digestive organs	1.81	0.37, 3.26	0.014
Malignant neoplasms of respiratory and intrathoracic organs	−0.13	−1.63, 1.38	0.870
Malignant neoplasms of the breast	3.01	1.48, 4.53	<0.001
Malignant neoplasms of female genital organs	1.79	0.20, 3.39	0.028
Malignant neoplasms, stated or presumed to be primary, of lymphoid, hematopoietic and related tissue	4.46	2.87, 6.05	<0.001
Year of catheterization			
2018	8.02	6.37, 9.67	<0.001
2019	10.26	8.98, 11.54	<0.001
2020	6.98	5.70, 8.25	<0.001
2021	5.15	3.91, 6.39	<0.001
2022	4.24	3.12, 5.36	<0.001
2023	−0.35	−1.39, 0.68	0.503
2024	1.03	0.17, 1.89	0.019
2025	Reference	-	-

Note: CI = confidence interval; B = unstandardized coefficient. To control for potential confounding bias, the effect estimates for each variable in the table have been mutually adjusted for all other covariates included in the model (sex, age group, side of placement, venipuncture site, tumor type, and year of placement). For each categorical variable, the most common clinical status or the most recent year was used as the reference category.

## Data Availability

The processed data used in this study are presented within the manuscript. Due to patient privacy concerns and ethical restrictions, the raw data cannot be publicly shared. Researchers may contact the first author or corresponding author upon reasonable request to obtain access to the data, subject to obtaining appropriate ethical approval.

## Supplementary Materials

The following supporting information can be downloaded at: https://www.mdpi.com/article/10.3390/healthcare14203442/s1, Table S1: Distribution of malignant tumor types among patients undergoing PICC placement; Table S2A: Complete pairwise comparisons of upper arm circumference among different diagnostic groups; Table S2B: Complete pairwise comparisons of external catheter length among different diagnostic groups; Table S2C: Complete pairwise comparisons of catheter insertion length among different diagnostic groups.

## Abbreviations

The following abbreviations are used in this manuscript:

PICC	Peripherally inserted central catheter
CVAD	Central vascular access device
CRBSIs	Catheter-related bloodstream infections
TPN	Total parenteral nutrition
ICD-10	International Statistical Classification of Diseases and Related Health Problems, 10th Revision
INS	Infusion Nurses Society
