# Supplementary material for: Demographic Characteristics and Evolution of Catheterization Patterns Among Cancer Patients with Peripherally Inserted Central Catheters in Huangshi, China: A Single-Center Retrospective Study
Source: Healthcare (Basel). 2026 Oct 14;14(20):3442. doi: 10.3390/healthcare14203442 (PMC13704004; doi:10.3390/healthcare14203442)
Supplement: Supplementary file 1 [file healthcare-14-03442-s001.zip › healthcare-4531456-supplementary.pdf]

Table S1: Distribution of malignant tumor types among patients undergoing PICC placement.

Distribution of Types of Malignant Neoplasms		
A: Malignant neoplasms of respiratory and intrathoracic organs	A1: Malignant neoplasm of thymus	n=1
	A2: Malignant neoplasm of larynx	n=9
	A3: Malignant neoplasm of lung	n=340
	A4: Malignant neoplasm of mediastinum	n=2
	A5:malignant neoplasm of middle ear	n=1
	A6: Malignant neoplasm of trachea	n=2
B: Malignant neoplasms of digestive organs	B1:Malignant neoplasm of rectum	n=279
	B2: Malignant neoplasm of colon	n=175
	B3: Malignant neoplasm of stomach	n=107
	B4: Malignant neoplasm of oesophagus	n=91
	B5: Malignant neoplasm of liver and intrahepatic bile ducts	n=1
	B6: Malignant neoplasm of intestine	n=2
	B7: Malignant neoplasm of liver	n=25
	B8: Malignant neoplasm of pancreas	n=28
	B9: Malignant neoplasm of small intestine	n=14
	B10: Malignant neoplasm of gallbladder	n=2
C: Malignant neoplasms of female genital organs	C1: Malignant neoplasm of ovary	n=77
	C2: Malignant neoplasm of fallopian tube	n=8
	C3: Malignant neoplasm of corpus uteri	n=60
	C4: Malignant neoplasm of cervix uteri	n=48
	C5: Malignant neoplasm of vulva	n=1
D: Malignant neoplasm of breast	D1: Malignant neoplasm of breast	n=282
	D2: Malignant lymphoma of breast	n=1
E: Malignant neoplasms, stated or presumed to be primary, of lymphoid, haematopoietic and related tissue	E1: Lymphoma	n=125
	E2: Malignant lymphoma	n=24
	E3: Leukaemia	n=7
	E4: Malignant plasma cell neoplasms	n=1
	E5: Malignant lymphoma of	n=1

	ovary	
	E6: Malignant lymphoma of breast	n=1
F: Other	F1: Malignant neoplasm of hypopharynx	n=1
	F2: Malignant neoplasm of nasopharynx	n=19
	F3: Malignant neoplasm of prostate	n=7
	F4: Malignant neoplasm of brain	n=2
	F5: Malignant neoplasm of bladder	n=2
	F6: Malignant neoplasm of thyroid gland	n=1
	F7: Malignant neoplasm of soft tissue	n=1
	F8: Malignant neoplasm of tongue	n=1
	F9: Malignant neoplasm of tonsil	n=1
	F10: Malignant neoplasm of adrenal gland	n=1
	F11: Malignant melanoma of skin	n=7
	F12: Malignant neoplasm of retroperitoneum and peritoneum	n=1
	F13: Malignant neoplasm of kidney	n=2

Table S2A. Complete pairwise comparisons of upper arm circumference among different diagnostic groups.

Comparison	Z	Adjusted p	Median diff. (95% CI)	Effect size (r)
Malignant neoplasms of respiratory and intrathoracic organs vs. Malignant neoplasms, stated or presumed to be primary, of lymphoid, haematopoietic and related tissue	-2.36	0.277	-1.00 (-1.00, 0.00)	-0.056
Malignant neoplasms of respiratory and intrathoracic organs vs. Malignant neoplasms of female genital organs	0.02	1.000	0.00 (-1.00, 0.00)	0.001
Malignant neoplasms of respiratory and intrathoracic organs vs. Other	-0.22	1.000	-1.00 (-1.00, 2.00)	-0.005
Malignant neoplasms of respiratory and intrathoracic organs vs. Malignant neoplasm of breast	-3.24	0.018	0.00 (-1.00, 0.50)	-0.077
Malignant neoplasms of respiratory and intrathoracic organs vs. Malignant neoplasms of digestive organs	-1.40	1.000	-1.00 (-1.00, 0.00)	-0.033
Malignant neoplasms, stated or presumed to be primary, of lymphoid, haematopoietic and related tissue vs. Malignant neoplasms of female genital organs	2.12	0.509	1.00 (-1.00, 1.00)	0.051

Malignant neoplasms, stated or presumed to be primary, of lymphoid, haematopoietic and related tissue vs. Other	1.14	1.000	0.00 (-1.00, 3.00)	0.027
Malignant neoplasms, stated or presumed to be primary, of lymphoid, haematopoietic and related tissue vs. Malignant neoplasm of breast	-0.34	1.000	1.00 (-1.00, 1.00)	-0.008
Malignant neoplasms, stated or presumed to be primary, of lymphoid, haematopoietic and related tissue vs. Malignant neoplasms of digestive organs	1.54	1.000	0.00 (-1.00, 1.00)	0.037
Malignant neoplasms of female genital organs vs. Other	-0.22	1.000	-1.00 (-1.00, 2.00)	-0.005
Malignant neoplasms of female genital organs vs. Malignant neoplasm of breast	-2.80	0.078	0.00 (-1.00, 1.00)	-0.067
Malignant neoplasms of female genital organs vs. Malignant neoplasms of digestive organs	-1.14	1.000	-1.00 (-1.00, 0.50)	-0.027
Other vs. Malignant neoplasm of breast	-1.41	1.000	1.00 (-2.00, 1.00)	-0.034
Other vs. Malignant neoplasms of digestive organs	-0.37	1.000	0.00 (-3.00, 1.00)	-0.009
Malignant neoplasm of breast vs. Malignant neoplasms of digestive organs	2.40	0.246	-1.00 (-1.00, 0.50)	0.057

Note: CI = confidence interval. Pairwise comparisons were performed using Dunn's test with Bonferroni correction. All pairwise comparison results are listed, including those with adjusted P ≥ 0.05.

Table S2B. Complete pairwise comparisons of external catheter length among different diagnostic groups.

Comparison	Z	Adjusted p	Median diff. (95% CI)	Effect size (r)
Malignant neoplasms of respiratory and intrathoracic organs vs. Malignant neoplasms, stated or presumed to be primary, of lymphoid, haematopoietic and related tissue	-0.62	1.000	0.00 (-1.00, 0.00)	-0.015
Malignant neoplasms of respiratory and intrathoracic organs vs. Malignant neoplasms of female genital organs	0.27	1.000	0.00 (0.00, 0.00)	0.006
Malignant neoplasms of respiratory and intrathoracic organs vs. Other	-0.22	1.000	0.00 (-1.00, 0.00)	-0.005
Malignant neoplasms of respiratory and intrathoracic organs vs. Malignant neoplasm of breast	-2.15	0.474	0.00 (-1.00, 0.00)	-0.051
Malignant neoplasms of respiratory and intrathoracic organs vs. Malignant neoplasms of digestive organs	-2.95	0.048	-1.00 (-1.00, -1.00)	-0.070
Malignant neoplasms, stated or presumed to be primary, of lymphoid, haematopoietic and related tissue vs.	0.78	1.000	0.00 (0.00, 1.00)	0.018

Malignant neoplasms of female genital organs				
Malignant neoplasms, stated or presumed to be primary, of lymphoid, haematopoietic and related tissue vs. Other	0.15	1.000	0.00 (-1.00, 1.00)	0.003
Malignant neoplasms, stated or presumed to be primary, of lymphoid, haematopoietic and related tissue vs. Malignant neoplasm of breast	-1.13	1.000	0.00 (-1.00, 1.00)	-0.027
Malignant neoplasms, stated or presumed to be primary, of lymphoid, haematopoietic and related tissue vs. Malignant neoplasms of digestive organs	-1.51	1.000	-1.00 (-1.00, 0.00)	-0.036
Malignant neoplasms of female genital organs vs. Other	-0.36	1.000	0.00 (-1.00, 0.00)	-0.008
Malignant neoplasms of female genital organs vs. Malignant neoplasm of breast	-2.10	0.542	0.00 (-1.00, 0.00)	-0.050
Malignant neoplasms of female genital organs vs. Malignant neoplasms of digestive organs	-2.66	0.118	-1.00 (-1.00, -1.00)	-0.063
Other vs. Malignant neoplasm of breast	-0.86	1.000	0.00 (-1.00, 1.00)	-0.021
Other vs. Malignant neoplasms of digestive organs	-1.03	1.000	-1.00 (-1.00, 0.00)	-0.025
Malignant neoplasm of breast vs. Malignant neoplasms of digestive organs	-0.28	1.000	-1.00 (-1.00, 0.00)	-0.007

Note: CI = confidence interval. Pairwise comparisons were performed using Dunn's test with Bonferroni correction. All pairwise comparison results are listed, including those with adjusted P ≥ 0.05.

Table S2C. Complete pairwise comparisons of catheter insertion length among different diagnostic groups.

Comparison	Z	Adjusted p	Median diff. (95% CI)	Effect size (r)
Malignant neoplasms of respiratory and intrathoracic organs vs. Malignant neoplasms, stated or presumed to be primary, of lymphoid, haematopoietic and related tissue	-8.10	< 0.001	-6.00 (-6.00, -4.00)	-0.193
Malignant neoplasms of respiratory and intrathoracic organs vs. Malignant neoplasms of female genital organs	-2.61	0.136	-4.00 (-4.00, -1.00)	-0.062
Malignant neoplasms of respiratory and intrathoracic organs vs. Other	-2.09	0.548	-4.00 (-5.00, 0.00)	-0.050
Malignant neoplasms of respiratory and intrathoracic organs vs. Malignant neoplasm of breast	-8.51	< 0.001	-6.00 (-6.00, -4.00)	-0.203
Malignant neoplasms of respiratory and intrathoracic organs vs. Malignant neoplasms of digestive organs	-9.63	< 0.001	-5.00 (-6.00, -3.00)	-0.229
Malignant neoplasms, stated or presumed to be primary,	5.04	< 0.001	2.00 (1.50,	0.120

of lymphoid, haematopoietic and related tissue vs. Malignant neoplasms of female genital organs			3.00)	
Malignant neoplasms, stated or presumed to be primary, of lymphoid, haematopoietic and related tissue vs. Other	2.66	0.118	2.00 (1.00, 6.00)	0.063
Malignant neoplasms, stated or presumed to be primary, of lymphoid, haematopoietic and related tissue vs. Malignant neoplasm of breast	0.95	1.000	0.00 (-1.00, 1.00)	0.023
Malignant neoplasms, stated or presumed to be primary, of lymphoid, haematopoietic and related tissue vs. Malignant neoplasms of digestive organs	1.70	1.000	1.00 (0.00, 1.00)	0.041
Malignant neoplasms of female genital organs vs. Other	-0.58	1.000	0.00 (-2.00, 4.00)	-0.014
Malignant neoplasms of female genital organs vs. Malignant neoplasm of breast	-4.78	< 0.001	-2.00 (-3.00, -1.00)	-0.114
Malignant neoplasms of female genital organs vs. Malignant neoplasms of digestive organs	-4.83	< 0.001	-1.00 (-2.00, -1.00)	-0.115
Other vs. Malignant neoplasm of breast	-2.21	0.409	-2.00 (-6.00, -1.00)	-0.053
Other vs. Malignant neoplasms of digestive organs	-1.95	0.773	-1.00 (-5.00, 0.00)	-0.046
Malignant neoplasm of breast vs. Malignant neoplasms of digestive organs	0.78	1.000	1.00 (0.00, 1.00)	0.019

Note: CI = confidence interval. Pairwise comparisons were performed using Dunn's test with Bonferroni correction. All pairwise comparison results are listed, including those with adjusted P ≥ 0.05.
